# Finding maximal exact matches in graphs

**DOI:** 10.1186/s13015-024-00255-5

**Published:** 2024-03-11

**Authors:** Nicola Rizzo, Manuel Cáceres, Veli Mäkinen

**Affiliations:** Department of Computer Science, University of Helsinki, Pietari Kalmin katu 5, P.O. Box 68, Helsinki, 00014 Finland

**Keywords:** Sequence to graph alignment, Bidirectional BWT, r-index, Suffix tree, Founder graphs

## Abstract

**Background:**

We study the problem of finding maximal exact matches (MEMs) between a query string *Q* and a labeled graph *G*. MEMs are an important class of seeds, often used in seed-chain-extend type of practical alignment methods because of their strong connections to classical metrics. A principled way to speed up chaining is to limit the number of MEMs by considering only MEMs of length at least $\kappa$ ($\kappa$-MEMs). However, on arbitrary input graphs, the problem of finding MEMs cannot be solved in truly sub-quadratic time under SETH (Equi et al., TALG 2023) even on acyclic graphs.

**Results:**

In this paper we show an $O(n\cdot L \cdot d^{L-1} + m + M_{\kappa ,L})$-time algorithm finding all $\kappa$-MEMs between *Q* and *G* spanning exactly *L* nodes in *G*, where *n* is the total length of node labels, *d* is the maximum degree of a node in *G*, $m = |Q|$, and $M_{\kappa ,L}$ is the number of output MEMs. We use this algorithm to develop a $\kappa$-MEM finding solution on indexable Elastic Founder Graphs (Equi et al., Algorithmica 2022) running in time $O(nH^2 + m + M_\kappa )$, where *H* is the maximum number of nodes in a block, and $M_\kappa$ is the total number of $\kappa$-MEMs. Our results generalize to the analysis of multiple query strings (MEMs between *G* and any of the strings). Additionally, we provide some experimental results showing that the number of graph MEMs is an order of magnitude smaller than the number of string MEMs of the corresponding concatenated collection.

**Conclusions:**

We show that seed-chain-extend type of alignment methods can be implemented on top of indexable Elastic Founder Graphs by providing an efficient way to produce the seeds between a set of queries and the graph. The code is available in https://github.com/algbio/efg-mems.

## Introduction

Sequence alignment has been studied since the 1970s [1, 2] and is a fundamental problem of computational molecular biology. Solving the classical problems of *longest common subsequence* (LCS) and *edit distance* (ED) between two strings takes quadratic time with simple dynamic programs, and it was recently proven that no strongly subquadratic-time algorithms exist conditioned on the Strong Exponential Time Hypothesis (SETH) [3, 4]. To overcome this hardness, researchers have used heuristics such as *co-linear chaining* [5]: by taking (short) matches between the input strings, known as *anchors*, we can take an ordered subset of these anchors and *chain* them together into an alignment. Furthermore, when using *maximal exact matches* (MEMs) as anchors, different co-linear chaining formulations capture both LCS [6] and ED [7] in near-linear time. MEMs are also used in popular seed-and-extend read aligners [8, 9]. In fact, practical tools limit the number of MEMs by considering only $\kappa$-MEMs (MEMs of length at least $\kappa$) [10, 11].

Extending alignment between sequences to sequence-to-graph alignment is an emerging and central challenge of *computational pangenomics* [12], as labeled graphs are a popular representation of pangenomes used in recent bioinformatics tools [13–16]. We assume that a labeled graph $G = (V,E,\ell )$ ($\ell :V \rightarrow \Sigma ^+$) is the reference pangenome of interest. Unfortunately, even finding exact occurrences of a given pattern in *G* does not admit strongly subquadratic-time solutions under SETH [17], and furthermore, a graph cannot be indexed in polynomial time to answer strongly subquadratic-time pattern matching queries [18]. To circumvent this difficulty, research efforts have concentrated on finding parameterized solutions to (exact) pattern matching in labeled graphs [19–22]. Moreover, the use of MEMs and co-linear chaining has also been extended to graphs [13–16].

In this paper, we study the problem of efficiently finding MEMs between a query string *Q* and a labeled graph *G*, where we extend the MEM definition to capture any maximal match between *Q* and the string spelled by some path of *G*. More precisely, our contributions are as follows:

- We adapt the MEM finding algorithm between two strings of Belazzougui et al. [23] to find all $\kappa$-node-MEMs between $Q$ and $G = (V,E,\ell )$ in $O(m + n + M_\kappa )$ time, where $m = |Q |$, $n = \sum _{v \in V} |\ell (v) |$ is the cumulative length of the node labels, and $M_\kappa$ is the number of $\kappa$-node-MEMs (of length at least $\kappa$ and between the node labels and $Q$).
- We extend the previous algorithm to find all $\kappa$-MEMs spanning exactly $L$ nodes of $G$ in time $O(m + n \cdot L \cdot d^{L-1} + M_{\kappa , L})$, where $d$ is the maximum degree of any node $v \in V$ and $M_{\kappa , L}$ are the $\kappa$-MEMs of interest. Note that MEMs spanning less than $L$ nodes can occur multiple times in paths spanning exactly $L$ nodes, and our contribution is to introduce an efficient technique to filter out these MEMs.
- Then we focus on graphs where constant $L$ is sufficient for $\kappa$-MEM finding:
  - We study $\kappa$-MEMs in indexable Elastic Founder Graphs (EFGs) [24], a subclass of labeled acyclic graphs admitting a poly-time indexing scheme for linear-time pattern matching. Given an indexable EFG $G$ of height $H$ (the maximum number of nodes in a graph block), we develop a suffix-tree-based solution to find all $\kappa$-MEMs spanning more than 3 nodes in $G$ in $O(nH^2 + m + M_{\kappa ,4+})$ time, where $M_{\kappa ,4+}$ are the number of output MEMs.
  - Combined with the above results for $L=1,2,3$, we can find $\kappa$-MEMs of an indexable EFG $G$ in $O(nH^2 + m + M_\kappa )$ time.
  - We note that the previous results easily generalize to the batched query setting: by substituting $Q$ with the concatenation of different query strings $Q_1$, ..., $Q_t$ of total length $m$, we compute all $\kappa$-MEMs between any query string and the graph with the same stated running time.
- Finally, we provide experimental results on finding MEMs from a collection of strains of SARS-CoV-2 and on an E. coli dataset. We use the bidirectional r-index [25] as the underlying machinery. On the one hand, we build the r-index of the concatenation of the covid19 strains and find all $m_{\kappa }$ $\kappa$-MEMs. On the other hand, we build an indexable EFG of the strains and find an upper bound on all $M_{\kappa }$ $\kappa$-MEMs in this case. On 100 strains, $M_{\kappa }$ is at least 34 times smaller than $m_{\kappa }$, thus confirming that graph MEMs compactly represent all MEMs. With the larger E. coli dataset, we study the indexing properties of our MEM finding approach. Experiments indicate that in practice MEM exploration depends linearly on the query length, while the theoretic worst case is quadratic.

The extensions to the conference version of this paper [26] consist of a) a more detailed description of the MEM finding algorithm (previous work and our adaptations) supported by pseudo-code and illustration, b) a study of the indexing setting, and c) a more engineered practical implementation as well as new experimental results.

## Preliminaries

### Strings

We denote integer intervals by [*x*..*y*], *x* and *y* inclusive. Let $\Sigma = [1..\sigma ]$ be an alphabet. A *string* *T*[1..*n*] is a sequence of symbols from $\Sigma$, that is, $T\in \Sigma ^n$ where $\Sigma ^n$ denotes the set of strings of length *n* over $\Sigma$. The *length* of a string *T* is denoted |*T*| and the *empty string* $\varepsilon$ is the string of length 0. In this paper, we assume that $|\Sigma |$ is constant. The concatenation of strings $T_1$ and $T_2$ is denoted as $T_1 \cdot T_2$, or just $T_1 T_2$. We denote by *T*[*x*..*y*] the *substring* of *T* made of the concatenation of its characters from the *x*-th to the *y*-th, both inclusive; if $x = y$ then we also use *T*[*x*] and if $y<x$ then $T[x..y] = \varepsilon$. The *reverse* of a string *T*[1..*n*], denoted by $\overline{T}$, is the string *T* read from right to left, that is, $\overline{T} = T[n]T[n-1]..T[1]$. A *suffix* (*prefix*) of string *T*[1..*n*] is *T*[*x*..*n*] (*T*[1..*y*]) for $1\le x \le n$ ($1 \le y \le n$) and we say it is *proper* if $x > 1$ ($y < n$). We denote by $\Sigma ^*$ the set of finite strings over $\Sigma$, and also $\Sigma ^+ = \Sigma ^*{\setminus } \{\varepsilon \}$. String *Q* *occurs* in *T* if $Q = T[x..y]$ for some interval [*x*..*y*]; in this case, we say that [*x*..*y*] is a match of *Q* in *T*. Moreover, we study matches between substrings of *Q* and *T*: a *maximal exact match* (MEM) between *Q* and *T* is a triplet $(x_1,x_2,\ell )$ such that $Q[x_1..\,x_1 + \ell - 1] = T[x_2..\,x_2 + \ell - 1]$ and the match cannot be extended to the left nor to the right, that is, $x_1 = 1$ or $x_2 = 1$ or $Q[x_1-1] \ne T[x_2-1]$ (*left-maximality*) and $x_1 + \ell = |Q |$ or $x_2 + \ell = |T |$ or $Q[x_1 + \ell ] \ne T[x_2 + \ell ]$ (*right-maximality*). In this case, we say that the substring $Q[x_1..\,x_1 + \ell - 1]$ is a *MEM string* between *Q* and *T*. The *lexicographic order* of two strings $T_1$ and $T_2$ is naturally defined by the total order $\le$ of the alphabet: $T_1 < T_2$ if and only if $T_1 \ne T_2$ and $T_1$ is a prefix of $T_2$ or there exists $y \ge 0$ such that $T_1[1..y]=T_2[1..y]$ and $T_1[y+1] < T_2[y+1]$. We avoid the prefix case by adding an *end marker* $\$\not \in \Sigma$ to the strings and considering $\$$ to be lexicographically smaller than any character in $\Sigma$.

### Labeled graphs

**Fig. 1:**
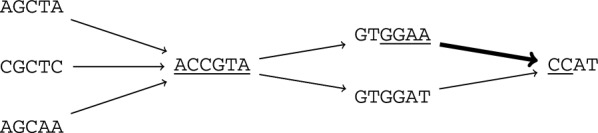
Substring $\texttt{ACCGTA}$ (underlined) with left-extension $\{\texttt{A},\texttt{C}\}$ and right-extension $\{\texttt{G}\}$, and substring GGAACC (underlined, bold edge) with left-extension $\{\texttt{T}\}$ and right-extension $\{\texttt{A}\}$

Let $G=(V,E,\ell )$ be a labeled graph with *V* being the set of nodes, *E* being the set of edges, and $\ell : V \rightarrow \Sigma ^+$ being a function giving a label to each node. A length-*k* *path* *P* from $v_1$ to $v_k$ is a sequence of nodes $v_1, \ldots , v_k$ connected by edges, that is, $(v_1,v_2),(v_2,v_3),\ldots ,(v_{k-1},v_k) \in E$. A node *u* *reaches* a node *v* if there is a path from *u* to *v*. The label $\ell (P) :=\ell (v_1) \cdots \ell (v_k)$ of *P* is the concatenation of the labels of the nodes in the path. For a node *v* and a path *P* we use $\Vert \cdot \Vert$ to denote its *string length*, that is, $\Vert v\Vert = |\ell (v)|$ and $\Vert P\Vert = |\ell (P)|$. Let *Q* be a query string. We say that *Q* *occurs* in *G* if *Q* occurs in $\ell (P)$ for some path *P*. In this case, the *exact match* of *Q* in *G* can be identified by the triple $(i, P = v_1\ldots v_k, j)$, where $Q = \ell (v_1)[i..] \cdot \ell (v_2) \cdots \ell (v_{k-1}) \cdot \ell (v_k)[..j]$, with $1 \le i \le \Vert v_1\Vert$ and $1 \le j \le \Vert v_k\Vert$, and we call such triple a *substring* of *G*. Given a substring (*i*, *P*, *j*) of *G*, we define its *left-extension* $\mathop {\text{lext}}\limits (i,P,j)$ as the singleton $\lbrace \ell (v_1)[i-1] \rbrace$ if $i > 1$ and otherwise as the set of characters $\lbrace \ell (u)[\Vert u\Vert ] \mid (u,v_1) \in E \rbrace$. Symmetrically, the *right-extension* $\mathop {\text{rext}}\limits (i,P,j)$ is $\lbrace \ell (v_k)[j+1] \rbrace$ if $j < \Vert v_k\Vert$ and otherwise it is $\lbrace \ell (v)[1] \mid (v_k, v) \in E \rbrace$. Note that the left (right) extension can be equal to the empty set $\emptyset$, if the start (end) node of *P* does not have incoming (outgoing) edges. Figure 1 illustrates these concepts.

### Basic tools

A *trie* or *keyword tree* [27] of a set of strings is an ordinal tree where the outgoing edges of each node are labeled by distinct symbols (the order of the children follows the order of the alphabet) and there is a unique root-to-leaf path spelling each string in the set; the shared part of two root-to-leaf paths spells the longest common prefix of the corresponding strings. In a *compact trie* [28], the maximal non-branching paths of a trie become edges labeled with the concatenation of labels on the path. The *suffix tree* of $T \in \Sigma ^*$ is the compact trie of all suffixes of the string $T' = T \$$ [29]. In this case, the edge labels are substrings of *T* and can be represented in constant space as an interval of *T*. Such a tree uses linear space and can be constructed in linear time, assuming that $|\Sigma |\le |T |$, so that when reading the root-to-leaf paths from left to right, the suffixes are listed in their lexicographic order [30, 31]. As such, the order spelled by the leaves of the suffix tree forms the *suffix array* $\textsf{SA} _T[1..|T'|]$, where $\textsf{SA} _T[i]=j$ iff $T'[j..|T'|]$ is the *i*-th smallest suffix in lexicographic order. When applied to a string *T*, the *Burrows–Wheeler transform* (BWT) [32] yields another string $\textsf{BWT} _T$ such that $\textsf{BWT} _T[i] = T'[\textsf{SA} [i] - 1]$ (we assume $T'$ to be a circular string, i.e. $T'[-1] = T'[|T| + 1] = \$$).

Let *Q*[1..*m*] be a query string. If *Q* occurs in *T*, then the *locus* or *implicit node* of *Q* in the suffix tree of *T* is (*v*, *k*) such that $Q = XY$, where *X* is the path spelled from the root to the parent of *v* and *Y* is the prefix of length *k* of the edge from the parent of *v* to *v*. The leaves in the subtree rooted at *v*, also known as *the leaves covered by v*, correspond to all the suffixes sharing the common prefix *Q*. Such leaves form an interval in the $\textsf{SA}$ and equivalently in the BWT. Let *aX* and *X* be the strings spelled from the root of the suffix tree to nodes *v* and *w*, respectively. Then one can store a *suffix link* from *v* to *w*. Suffix links from implicit nodes are called implicit suffix links.

The *bidirectional BWT of T* [33], that we denote as $\texttt{idx}_T$, is a compact BWT-based index capable of performing a text search in $T'$ in both directions while synchronizing the intervals of the searched string in $\textsf{SA} _{T}$ and $\textsf{SA} _{\overline{T}}$. In the following, we assume $T'$ to be circular, thus the character preceding $T'[1]$ is $\$$ and the one following $T'[|T' |]$ is *T*[1]. The index supports the following operations, given interval $[i..j]$ in $\textsf{SA} _T$ and interval $[i'\!..j']$ in $\textsf{SA} _{\overline{T}}$ representing a substring $Q'$ of *T*: $\texttt{idx}_T.\texttt{isLeftMaximal}(i,j)$ ($\texttt{isRightMaximal}(i',j')$) returns false if and only if all occurrences of $Q'$ in *T* are preceded (followed) by the same character, and true otherwise, in which case we say that $Q'$ is a left-maximal (right-maximal) string of *T*; $\texttt{idx}_T.\texttt{enumerateLeft}(i,j)$ ($\texttt{enumerateRight}(i',j')$) returns all distinct characters that precede (follow) the occurrences of $Q'$ in *T*; and, given $c \in \Sigma \cup \lbrace \$ \rbrace$, $\texttt{idx}_T.\texttt{extendLeft}(c,[i..j],[i'\!..j'])$ ($\texttt{extendRight}(c,[i..j],[i'\!..j'])$) returns the pair of intervals $[x..y]$, $[x'\!..y']$ of $\textsf{SA} _T$ and $\textsf{SA} _{\overline{T}}$, respectively, representing the substring $cQ'$ ($Q'c$) of *T*. Since we assume $|\Sigma |\in O(1)$, index $\texttt{idx}_T$ can be built in $O(|T |)$ time to support the presented operations in output-sensitive time, that is, linear in the size of the output [33]. The bidirectional BWT of *T* is capable of simulating the navigation of the suffix tree nodes of *T* in $O(|T |)$ time. Moreover, Belazzougui et al. [23] show how to solve the MEM finding problem in linear time: their algorithm uses the indices $\texttt{idx}_Q$ and $\texttt{idx}_T$ and simulates the traversal of the common strings in the suffix tree of $Q \# T$, with $\# \notin \Sigma$. When this traversal finds a candidate MEM string, the algorithm outputs all the corresponding MEMs in output-sensitive time by using a cross-product routine. We present a detailed explanation of this solution in the next section.

Let *B*[1..*n*] be a bitvector, that is, a string over the alphabet $\{0,1\}$. There is a data structure that can be constructed in time *O*(*n*) which answers $r = \texttt{rank}(B,i)$ and $j=\texttt{select}(B, r)$ in constant time, where the former operation returns the number of 1s in *B*[1..*i*] and the latter returns the position $j\le i$ of the *r*-th 1 in *B* [34, 35].

Let *D*[1..*n*] be an array of integers. There is a *range minimum query* data structure that can be constructed in *O*(*n*) time which answers $\textsf {RMQ} _D(i,j)$ in constant time [36], where $\textsf {RMQ} _D(i,j)$ returns an index *k* such that *D*[*k*] is the minimum value in the subarray *D*[*i*..*j*]. We will use the following lemma that exploits range queries recursively.

#### Lemma 1

Let *D*[1..*n*] be an array of integers. One can preprocess *D* in *O*(*n*) time so that given a threshold $\Delta$, one can list all elements of *D* such that $D[i]\le \Delta$ in linear time in the size of the output.

#### Proof

We can build the range minimum query data structure on *D*. Consider a recursive algorithm analogous to the one in [37] which starts with $k=\textsf {RMQ} _D(1,n)$. If $D[k] > \Delta$, the algorithm stops as no element in the range is at least $\Delta$. Otherwise, the algorithm reports *k* and recursively continues with $\textsf {RMQ} _D(1,k-1)$ and $\textsf {RMQ} _D(k+1,n)$. Note that each recursive call performs exactly one $\textsf {RMQ}$ operation: if an element is reported the $\textsf {RMQ}$ is charged to this element, otherwise it is charged to its parent (in the recursion tree), and thus the number of $\textsf {RMQ}$ operations is linear in the output size.

## Finding MEMs in labeled graphs

Let us consider the problem of finding all *maximal exact matches* (MEMs) between a labeled graph $G=(V,E,\ell )$, with $\ell : V \rightarrow \Sigma ^+$, and a query string $Q \in \Sigma ^+$.

### Definition 1

(MEM between a pattern and a graph) Let $G = (V,E,\ell )$ be a labeled graph, with $\ell :V \rightarrow \Sigma ^+$, let $Q \in \Sigma ^+$ be a query string, and let $\kappa >0$ be a threshold. Given a match (*i*, *P*, *j*) of *Q*[*x*..*y*] in *G*, we say that the pair ([*x*..*y*], (*i*, *P*, *j*)) is *left-maximal* (*right-maximal*) if it *cannot* be extended to the left (right, respectively) in both *Q* and *G*, that is,

$$\begin{aligned} (\textsf{LeftMax})&\quad x=1 \;\vee \; \mathop {\text{lext}}\limits (i,P,j) = \emptyset \;\;\vee \\ & \quad\!\vee\; Q[x-1] \notin \mathop {\text{lext}}\limits (i,P,j) \qquad \text {and}\\ (\textsf{RightMax})&\quad y = |Q |\;\vee \; \mathop {\text{rext}}\limits (i,P,j) = \emptyset \;\;\vee \\ & \quad\!\vee\; Q[y+1] \notin \mathop {\text{rext}}\limits (i,P,j). \end{aligned}$$

We call ([*x*...*y*], (*i*, *P*, *j*)) a $\kappa$-MEM iff $\textsf{LeftMax} \vee |\mathop {\text{lext}}\limits (i,P,j) |\ge 2$, $\textsf{RightMax} \vee |\mathop {\text{rext}}\limits (i,P,j) |\ge 2$, and $y-x+1\ge \kappa$, meaning that it is of length at least $\kappa$, it is left-maximal or its left (graph) extension is not a singleton, and it is right-maximal or its right (graph) extension is not a singleton.

We use this particular extension of MEMs to graphs—with the additional conditions on non-singletons $\mathop {\text{lext}}\limits$ and $\mathop {\text{rext}}\limits$—as it captures all MEMs between *Q* and $\ell (P)$, where *P* is a source-to-sink path in *G*. Indeed, note that removing the non-singleton conditions would miss matches that can be extended through one path but not another. For example, with $Q=\texttt{CACCGTAT}$, $\kappa =0$, *v* being the first underlined node of Fig. 1, and *u* being the second in-neighbor of *v*, then ([1..7], (5, *uv*, 6)) is a MEM since it is left and right maximal. Note that pair ([2..7], (1, *v*, 6)) is also a MEM since it is right-maximal, and the left extension of (1, *v*, 6) is not a singleton ($\mathop {\text{lext}}\limits (v) = \lbrace \texttt{A}, \texttt{C} \rbrace$): this match is not left-maximal but our definition includes it as there are at least two different characters to the left. Moreover, this MEM formulation (with $\kappa =1$) captures LCS through co-linear chaining, whereas avoiding the additional conditions would fail [38].

The rest of this section is structured as follows. First, we show how to adapt the MEM finding algorithm of Belazzougui et al. [23] for the case of node $\kappa$-MEMs, which ignore the singleton conditions of Definition 1. Then, we show how to further generalize this approach to report all $\kappa$-MEMs spanning exactly *L* nodes.

### MEMs in node labels

We say that a match ([*x*..*y*], (*i*, *P*, *j*)) is a *node MEM* if *P* is a path of length 1, i.e. $P = v$ for some node *v*, and the match is left and right maximal w.r.t. $\ell (P)$ only in the string sense. In other words, a node MEM is a (string) MEM between *Q* and $\ell (v)$ (especially in the case $x=1$ or $y=\ell (v)$). For this, we consider the text

$$\begin{aligned} T_\text {nodes} = \textbf{0} \cdot \prod _{v \in V} \Big ( \ell (v) \cdot \textbf{0} \Big ), \end{aligned}$$

where $\textbf{0}\notin \Sigma$ is used as a delimiter to prevent MEMs spanning more than a node label.

Running the MEM finding algorithm of Belazzougui et al. [23, Theorem 7.10] on *Q* and $T_\text {nodes}$ will retrieve exactly the node MEMs we are looking for. Given such a MEM $(x_1, x_2, \ell )$, to transform the coordinates of $T_\text {nodes}[x_2..\,x_2+\ell -1]$ into the corresponding graph substring (*i*, *P*, *j*) we augment the index with a bitvector *B* marking the locations of $\textbf{0}$s of $T_\text {nodes}$, so that $r = \texttt{rank}(B,x_2)$ identifies the corresponding node of *G*, $i =x_2-\texttt{select}(B, r)$ and $j =i+\ell -1$. The following result follows directly.

**Algorithm 1:**
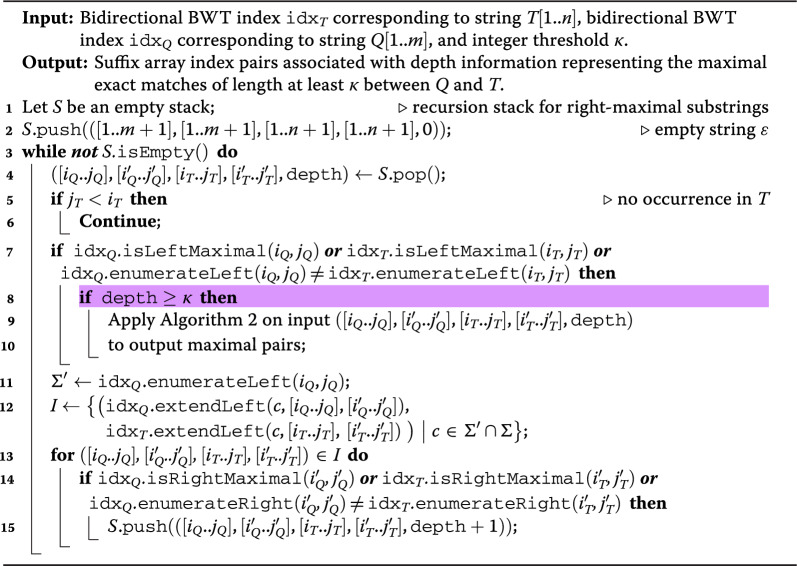
Computing a representation of $\kappa$-MEMs between *Q* and $T = T_\text{nodes}$ using their bidirectional BWT indexes. The algorithm explores all MEM candidates and it calls Algorithm 2 to output the MEMs.

**Algorithm 2:**
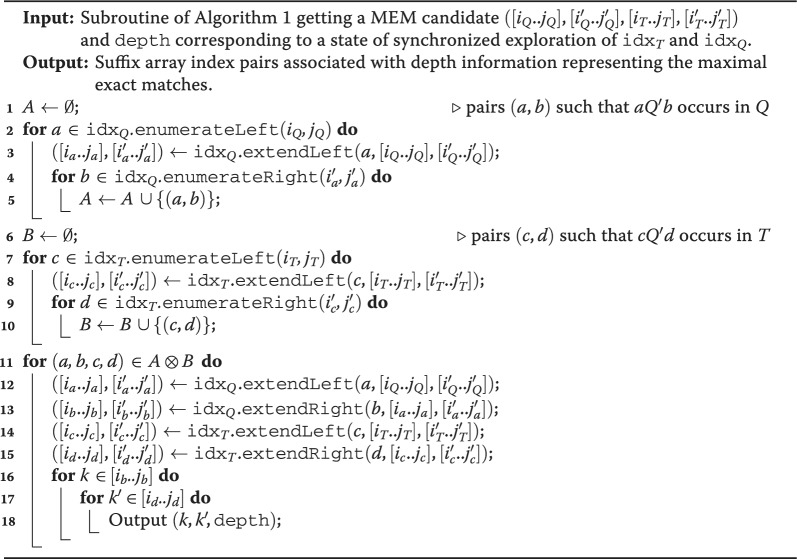
Cross-product computation for outputting a representation of maximal exact matches using two bidirectional BWT indexes. The input is a MEM candidate computed in Algorithm 1. Here $(a,b,c,d) \in A \otimes B$ iff $(a,b) \in A$, $(c,d) \in B$, $a\ne c$, and $b\ne d$.

#### Lemma 2

Let alphabet $\Sigma$ be of constant size. Given a labeled graph $G = (V,E,\ell )$, with $\ell : V \rightarrow \Sigma ^+$, a query string *Q*, and a threshold $\kappa >0$, we can compute all node MEMs of length at least $\kappa$ between *Q* and *G* in time $O(n+m+N_\kappa )$, where *n* is the total length of node labels, $m = |Q|$, and $N_\kappa$ is the number of output MEMs.

As we will modify this algorithm later on, we now describe a self-contained and simplified version of the MEM finding algorithm of [23, Theorem 7.10] sufficient for our purposes as Algorithms 1 and 2. This version closely follows the one in the textbook [29, Algorithms 18 and 19], with the simple modification of computing only MEMs of length at least $\kappa$ (highlighted in Algorithm 1). Moreover, we discuss the differences from the original approach at the end of this section.

Let $T = T_\text{nodes}$. We say that a string $Q'$ is a *MEM candidate* if $|Q' |\ge \kappa$, $Q'$ is left- and right-maximal in $T\#Q$, and it occurs in both *T* and *Q*. Algorithm 1 uses $\texttt{idx}_{T}$ and $\texttt{idx}_{Q}$, supporting the operations described in the preliminaries, to recursively consider all right-maximal substrings of $T\#Q$ that occur in both *T* and *Q*. Then, for the subset of these substrings that are MEM candidates, it calls a *cross-product* routine, Algorithm 2, outputting all MEMs whose MEM string is $Q'$.

**Fig. 2:**
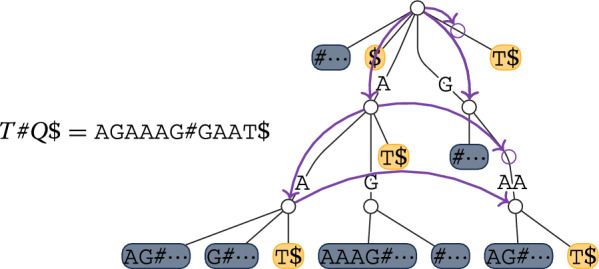
Representation of the suffix tree of $T\#Q$, with $T = \texttt{AGAAAG}$ and $Q = \texttt{GAAT}$, where leaves corresponding to suffixes starting inside *T* and *Q* are marked in blue and orange, respectively. The colored arrows follow the traversal simulated by Algorithm 1: the recursion stops when the considered substring of *Q* does not occur in *T* (line 5), as is the case for $Q[4] = \texttt{T}$, or when the same character follows all of its occurrences in both *Q* and *T* (line 13), as is the case for $Q[1..2] = T[2..3] = \texttt{GA}$

To see the linearity with respect to the input strings, let us study how the MEM candidates $Q'$ are explored. The algorithm starts with intervals of $\texttt{idx}_Q$ and $\texttt{idx}_T$ corresponding to the empty string $\varepsilon$ (line 2). By using stack *S*, it then considers recursively all strings $Q'$ that occur in both *Q* and *T* and that are right-maximal in $T\#Q$ by extending the currently matched $Q'$ to the left with all possible extensions in *Q* (lines 10-11). The recursion continues only if the extended string is still right-maximal (line 13) and occurs in *T* (line 5). See Fig. 2 for a visual example in the corresponding suffix tree of $T\#Q$. When a considered right-maximal $Q'$ is of length at least $\kappa$ and is also left-maximal in $Q\#T$ (lines 7–8), thus it is a MEM candidate, Algorithm 2 is called to report the corresponding MEMs: it implements a cross-product routine considering the occurrences of $aQ'b$ in *Q* (lines 1–5) and of $cQ'd$ in *T* (lines 6–10) such that $a \ne c$ and $b \ne d$ (lines 11–18). In other words, if *A* is the set or pairs (*a*, *b*) such that $aQ'b$ occurs in *Q* and *B* is the set of pairs (*c*, *d*) such that $cQ'd$ occurs in *T*, it has to consider the cross product $A \otimes B$, defined as the set of tuples (*a*, *b*, *c*, *d*) such that $(a,b) \in A$, $(c,d) \in B$, and $a \ne c$ and $b \ne d$.

Recall that operations on $\texttt{idx}_Q$ and $\texttt{idx}_T$ take constant time. The time complexity of Algorithm 1 is $O(|T |+ |Q |+ N_\kappa )$. The exploration performed by Algorithm 1 takes constant time per right-maximal substring of $T\#Q$: the right-maximal substrings are the suffix tree nodes of $T\#Q$, and the number of left extensions where the recursion does not continue is at most the number of right-maximal substrings multiplied by a constant alphabet factor. Moreover, since $\Sigma$ has constant size, the calls to Algorithm 2 globally consider at most $|\Sigma ^2 |\in O(1)$ extensions for each MEM candidate $Q'$ and they output all $N_\kappa$ MEMs in $O(|T |+ |Q |+ N_\kappa )$ time, reaching the stated time complexity.

Finally, note that Algorithms 1 and 2 output the representation of MEMs using their positions in $\textsf{SA} _{Q}$ and $\textsf{SA} _{T}$, instead of their position in the text. More precisely, these positions refer to an occurrence of $aQ'b$ in *Q* and $cQ'd$ in *T* such that $a \ne c$ and $b \ne d$ and each triple $(k,k',\ell )$ in this format corresponds to MEM $(x,y,\ell )$ between *Q* and *T* such that $x = \textsf{SA} _Q[k] + 1$ and $y = \textsf{SA} _T[k'] + 1$. This conversion can be executed in $O(|T |+ |Q |)$ time by simply computing $\textsf{SA} _Q$ and $\textsf{SA} _T$ and retrieving *x* and *y* as described above. Alternatively, the original algorithm proposes a batched locate query solution that, in our setting, works in $O(|T |+ |Q |+ N_\kappa )$ time and has working space complexity of $O(N_\kappa ( \log |T |+ \log |Q |))$ bits [23, Lemma 3.1].

Our description differs from the original [23] as follows:

- we assume that alphabet $\Sigma$ has constant size, instead of assumption $|\Sigma |\in o(\sqrt{|T |} / \log |T |)$;
- we use the bidirectional BWT index as the representation of $T$ and $Q$, while the original uses a unidirectional BWT index to avoid randomized construction time for larger alphabets;
- our simplified complexity analysis works for a constant-sized alphabet, but the authors prove—with a more sophisticated analysis—that Algorithms 1 and 2 take time $O(|T |+ |Q |+ N_\kappa )$, when $|\Sigma |\in o(\sqrt{|T |} / \log |T |)$;
- the original algorithm, when branching in the exploration of the suffix tree of $T\# Q$, explores first the branch with the most occurrences in $T$ or $Q$, to claim the working space complexity of $O((|T |+ |Q |) \cdot \log |\Sigma |)$ bits.

Our later modifications assume a constant-sized alphabet and use more working space, thus our analysis and the simplified exploration of Algorithm 1 are sufficient.

### MEMs spanning exactly *L* nodes

Given a threshold $\kappa$, we want to find all $\kappa$-MEMs ([*x*...*y*], (*i*, *P*, *j*)) spanning exactly *L* nodes in *G*, that is, $|P |= L$. Note that the MEMs obtained for $L=1$ are a subset of the node MEMs obtained in Lemma 2: for a node MEM ([*x*...*y*], (*i*, *v*, *j*)) it might hold that $i = 1$ and $\lbrace Q[x-1] \rbrace = \mathop {\text{lext}}\limits (1,v,j)$, or that $j = \Vert v\Vert$ and $\lbrace Q[y+1] \rbrace = \mathop {\text{rext}}\limits (i,v,j)$. Indeed, as per Definition 1, MEMs cannot be recognized without looking at the context of the paths in *G* (sets $\mathop {\text{lext}}\limits$ and $\mathop {\text{rext}}\limits$). With this in mind, we consider the text

1 $$\begin{aligned} T_L :=\textbf{0} \cdot \prod _{(u_1, \dots , u_L) \in \mathcal {P}_G^L} \Big (\mathop {\text{left}}\limits (u_1) \cdot \ell (u_1) \cdots \ell (u_L) \cdot \mathop {\text{right}}\limits (u_L) \cdot \textbf{0} \Big ), \end{aligned}$$

where $\mathop {\text{left}}\limits (u)=c$ when $\mathop {\text{lext}}\limits (u)=\{c\}$ and otherwise $\mathop {\text{left}}\limits (u)=\#$, $\mathop {\text{right}}\limits (u)=d$ when $\mathop {\text{rext}}\limits (u)=\{d\}$ and otherwise $\mathop {\text{right}}\limits (u)=\#$, with $\textbf{0},\# \notin \Sigma$ two distinct characters and

$$\begin{aligned} \mathcal {P}^L_G :=\bigg \lbrace P \biggm \vert \begin{array}{c} \displaystyle P \;\text {path of}\; G, \\ \displaystyle |P |= L \end{array} \bigg \rbrace . \end{aligned}$$

We have added the unique left- and right-extension symbols *c* and *d* to avoid reporting exact matches that can potentially be extended to longer paths. When these extensions are not unique (or empty), one can safely report a MEM, since there is a path diverting with a symbol different from that of the pattern (or the path cannot be extended further). In addition to these left- and right-extension symbols, we modify the MEM finding algorithm designed for node MEMs to use some extra information regarding the starting position of each suffix inside string $\ell (P)$, as explained next.

To avoid reporting MEMs spanning less than *L* nodes (only if $L > 1$), we use an array $D[1..|T_L|]$ such that $D[k]=\infty$ if the *k*-th suffix $T_L[s..|T_L|]$ of $T_L$ in the lexicographic order is such that $T_L[s+1..|T_L|]$ is not starting inside node $u_1$ of a path $P=u_1 \cdots u_L$, otherwise $D[k]=|\ell (P)|-\ell (u_L)-i+2$, where suffix $T_L[s+1..|T_L|]$ starts at position *i* inside $u_1$. That is, when $D[k]\ne \infty$, it tells the distance of the *k*-th suffix of $T_L$ in the lexicographic order to the start of the last node of the corresponding path. With the help of Lemma 1 on *D*, we can then adapt the MEM finding algorithm to output suffixes corresponding to MEMs spanning exactly *L* nodes as follows.

#### Lemma 3

Let alphabet $\Sigma$ be of constant size. Given a labeled graph $G = (V,E,\ell )$, a pattern $Q \in \Sigma ^m$, a threshold $\kappa \ge 1$, and an integer $L \ge 1$, we can compute all $\kappa$-MEMs between *Q* and *G* spanning exactly *L* nodes of *G* in time $O(m + |T_L |+ M_{\kappa ,L})$. Here, $T_L$ is defined as in Equation (1) and $M_{\kappa ,L}$ is the number of output MEMs.

#### Proof

We build the bidirectional BWT indexes $\texttt{idx}_{T_L}$ and $\texttt{idx}_Q$ and the suffix arrays $\textsf{SA} _{T_L}$ and $\textsf{SA} _Q$ for $T_L$ and *Q*, respectively, and preprocess $D[1..|T_L|]$ as in Lemma 1 in time $O(|T_L |+|Q |)$. We also preprocess, in linear time, a bitvector *B* marking the locations of $\textbf{0}$s of $T_L$ so that we can map in constant time a position *i* in $T_L$ to the *r*-th path appended to $T_L$ for $r = \texttt{rank}(B,i)$.

The modifications to Algorithms 1 and 2 required to only output an encoding of MEMs between *Q* and *G* spanning exactly *L* nodes (and only if $L>1$) is to change its last steps in Algorithm 2 when considering a MEM candidate $Q'$. We present such modifications in Algorithm 3 (highlighted lines). Namely, the cross-product routine loops over all characters $a,b \in \Sigma \cup \{\#,\$\}$ and $c,d \in \Sigma \cup \{\#\}$ with $a\ne c$ and $b\ne d$, such that $aQ'b$ is a substring of *Q* and $cQ'd$ is a substring of $T_L$. It then computes (in constant time) the intervals $[i_{aQ'b}..j_{aQ'b}]$, $[i'_{aQ'b}..j'_{aQ'b}]$, $[i_{cQ'd}..j_{cQ'd}]$, and $[i'_{cQ'd}..j'_{cQ'd}]$, where the first two are the intervals in the bidirectional BWT on *Q* corresponding to $aQ'b$ and the latter two are the intervals in the bidirectional BWT on $T_L$ corresponding to $cQ'd$. Note that the modification to the computation of set *B* in Algorithm 3 avoids extensions with $\textbf{0}$ in lines 3 and 5. Finally, the algorithm outputs a triple $(k',k,|Q'|)$ representing each MEM, where $k\in [i_{cQ'd}..j_{cQ'd}]$ and $k' \in [i_{aQ'b}..j_{aQ'b}]$. Here it suffices to modify the first iteration using Lemma 1 to loop only over $k \in [i_{cQ'd}..j_{cQ'd}]$ such that $D[k]\le |Q'|+1$ (line 12).

Our claims are that the running time stays linear in the input and output size on constant-size alphabet and that only MEMs spanning exactly *L* nodes are output. The latter claim follows directly on how array *D* is defined and used with Lemma 1. For the former claim, the cross-product part of the original algorithm is linear in the output size (also on non-constant-size alphabet) since for each combination of left- and right-extension considered, the work can be charged to the output. In our case, due to the use of Lemma 1, some combinations may lead to empty outputs introducing an alphabet-factor (constant) multiplier on the input length.

**Algorithm 3:**
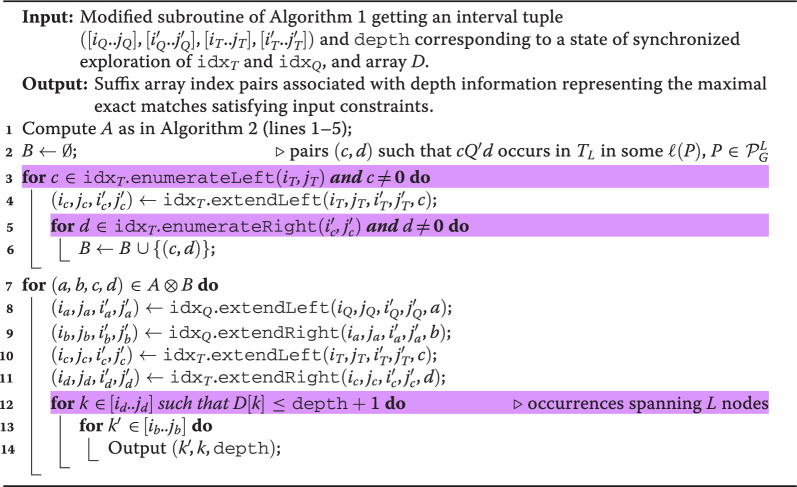
Constrained cross-product computation for outputting maximal exact matches using two bidirectional BWT indexes

#### Remark 1

Note that the algorithm in Lemma 3 works in time $O(m + n \cdot L \cdot d^{L-1} + M_{\kappa ,L})$, where $n = \sum _{v \in V} \ell (v)$ is the total label length of *G* and *d* is the maximum in- or out-degree of a node. Indeed, $T_{L}$ corresponds to the concatenation of length-*L* paths of *G*: the number of paths containing label $\ell (v)$ (for a node *v*) is at most $L\cdot d^{L-1}$.

## MEMs in elastic founder graphs

Recall that exact pattern matching in labeled graphs does not admit a strongly subquadratic-time solution. More specifically, Equi et al. proved that answering whether a pattern *Q* occurs in a labeled graph $G=(V,E,\ell )$ cannot be answered in $O(m^{1 - \varepsilon } |E |)$ or $O(m |E |^{1 - \varepsilon })$ time for any constant $\varepsilon > 0$ under the Strong Exponential Time Hypothesis (SETH), even if *G* is acyclic [17]^1^. It is easy to see that this hardness extends to $\kappa$-MEM finding between *Q* and *G*: if $\kappa = |Q |$, the $\kappa$-MEMs between *Q* and *G* are exactly the occurrences of *Q* in *G*. As shown in Remark 1, the approach of Lemma 3 to find MEMs spanning *L* nodes in *G* is exponential on *L* and it is unfeasible for large values of *L*. To have an efficient solution for MEM finding, we restrict the family of graphs that we consider to that of indexable Elastic Founder Graphs (indexable EFGs), which are a subclass of labeled directed acyclic graphs (labeled DAGs) that admit exact pattern matching solutions breaking through the quadratic-time lower bounds [24]. In this section, we show that Lemma 3, together with the extension of the techniques used to query if *Q* appears in an indexable EFG *G*, can solve $\kappa$-MEM finding in *G* in parameterized linear time.

### Definition 2

(Elastic Founder Graph [24, 39]) Consider a *block graph* $G = (V,E,\ell )$, where $\ell :V \rightarrow \Sigma ^+$, *V* is partitioned into *k* *blocks* $V_1$, ..., $V_k$, and edges $(u,v) \in E$ are such that $u \in V_i$, $v \in V_{i+1}$ for some $i \in [1..k-1]$. We say that a block graph is an *indexable Elastic Founder Graph* (indexable EFG) if the *semi-repeat-free property* holds: each $\ell (v)$ for $v\in V$ occurs in *G* only starting from the beginning of $w\in V$, where *w* is from the same block as *v*.

Note that the semi-repeat-free property allows a node label to be a prefix of other node labels in the same block, whereas it forbids them to appear as a proper suffix of other node labels or anywhere else in the graph. Indexable EFGs can be obtained from a set of aligned sequences, in a way such that the resulting indexable EFG spells the sequences but also their *recombination*: for a gapless alignment, we can build in time linear to the size of the alignment an optimal indexable EFG with minimum height *H* of a block, where the height of block $V_i$ is defined as $|V_i |$, solution generalized to the case with gaps by using an alternative height definition [40].

Let us now consider MEM finding with threshold $\kappa$ on an indexable EFG $G = (V,E,\ell )$. We can use the general $\kappa$-MEM finding algorithm of Lemma 3 between *Q* and *G* spanning exactly *L* nodes, with $L = 1,2,3$; then, we find $\kappa$-MEMs that span longer paths with a solution specific to indexable EFGs. To find all MEMs between *Q* and *G* spanning more than three nodes, we index

$$\begin{aligned} T_3' :=\textbf{0} \cdot \prod _{(u,v),(v,w) \in E} \Big ( \ell (u) \cdot \ell (v) \cdot \ell (w)\cdot \textbf{0} \Big ) \qquad \text {where}\; \textbf{0} \notin \Sigma . \end{aligned}$$

Equi et al. [24] showed that the suffix tree of $T_3'$ can be used to query string *Q* in *G*, taking time $O(|Q |)$. We now extend this algorithm to find MEMs between *Q* and indexable EFG *G* with threshold $\kappa$ and spanning more than 3 nodes. For simplicity, we describe a solution for case $\kappa = 1$ and later argue case $\kappa > 1$.

First, we augment the suffix tree of $T_3'$:

- we mark all implicit or explicit nodes $\overline{p}$ such that the corresponding root-to-$\overline{p}$ path spells $\ell (u)\ell (v)$ for some $(u,v) \in E$, so that we can query in constant time if $\overline{p}$ is such a node;
- we compute pointers from each node $\overline{p}$ to an arbitrarily chosen leaf in the subtree rooted at $\overline{p}$;
- for each node $v \in V$ of the indexable EFG we build trie $T_v$ for the set of strings $\lbrace \overline{\ell (u)}: (u,v) \in E \rbrace$;
- for each leaf, we store the corresponding path *uvw* and the starting position of the suffix inside $\ell (u)\ell (v)\ell (w)$.

### Observation 1

([24, Lemma 9]) Given an indexable EFG $G=(V,E,\ell )$, for each $(v,w) \in E$ string $\ell (v)\ell (w)$ occurs only as prefix of paths starting with *v*. Thus, all occurrences of some string *S* in *G* spanning at least four nodes can be decomposed as $\alpha \ell (u_2) \cdots \ell (u_{L-1}) \beta$ such that: (*i*) $u_2 \cdots u_{L-1}$ is a path in *G* and $u_2$, ..., $u_{L-1}$ are unequivocally identified; (*ii*) $\alpha = \ell (u_1)[i..\Vert u_1 \Vert ]$ with $1 \le i \le \Vert u_1 \Vert$ for some $(u_1,u_2) \in E$; and (*iii*) $\beta = \ell (u_L)$ for some $(u_{L-1},u_L) \in E$ or $\beta = \ell (u_L) (\ell (u_{L+1})[1..j])$ with $1 \le j < \Vert u_{L+1} \Vert$ for some $(u_{L-1},u_L),(u_L,u_{L+1}) \in E$. Note that $\alpha ,\beta \ne \varepsilon$ and $\beta$ has as prefix a full node label, whereas $\alpha$ might spell any suffix of a node label.

The strategy to compute long MEMs between *Q* and *G* is to first consider, with a left-to-right scan of *Q*, all MEMs ([*x*..*y*], (*i*, *P*, *j*)) such that:

I. $$|P |> 3$$;
II. they satisfy conditions $\textsf{LeftMax}$ and $\textsf{RightMax}$ of Definition 1; and
III. are maximal with respect to substring *Q*[*x*..*y*], that is, there is no other MEM $([x'\!..y'],(i',P',j'))$ with $x \le x' \le y' \le y$.

Next, we will describe how to modify our solution to compute all the other MEMs spanning more than 3 nodes. Due to Observation 1, if $\alpha \ell (u_2) \cdots \ell (u_{L-1}) \beta$ is a decomposition of *Q*[*x*..*y*], all MEMs $([x'\!..y'],(i',P',j'))$ with $x \le x' < y' \le y$ spanning more than 3 nodes are constrained to involve some $u_i$ with $i \in [2..L-1]$.

Consider the following modification of [24, Theorem 8] that matches *Q*[1..*y*] in *G*. Let $\overline{p}$ be the suffix tree node of $T_3'$ reached from the root by spelling *Q*[1..*y*] in the suffix tree until we cannot continue with $Q[y+1]$:

1. If we cannot continue with $\textbf{0}$, $Q$[1..*y*] is part of some MEM between $Q$ and $G$ spanning at most 3 nodes, so we ignore it, take the suffix link of $\overline{p}$ and consider matching $Q[2..y]$ in $G$.
2. If we can continue with $\textbf{0}$ and the occurrences of $Q[1..y]$ span at most two nodes in $G$, then we also take the suffix link of $\overline{p}$ and consider matching $Q[2..y]$. Thanks to the semi-repeat-free property, we can check this condition by retrieving any leaf in the subtree rooted at node $\overline{p}_\textbf{0}$, reached by reading $\textbf{0}$ from $\overline{p}$.
3. In the remaining case, $Q[1..y] = \alpha \ell (u_2) \ell (u_3)$ for exactly one $u_2 \in V$, with $(u_2,u_3) \in E$, due to Observation 1, and we follow the suffix link walk from $\overline{p}$ until we find the marked node $\overline{q}$ corresponding to $\ell (u_2) \ell (u_3)$: from $\overline{q}$ we try to match $Q[y+1..]$ until failure, matching $Q[y+1..y']$ and reaching node $\overline{r}$.

By repeating the suffix walk and tentative match of case 3 until we cannot read $\textbf{0}$ from the failing node, we find the maximal prefix *Q*[1..*y*] occurring in *G* and its decomposition $\alpha \ell (u_2) \cdots \ell (u_{L-1}) \beta$ as per Observation 1. Indeed, we can find unique nodes $u_2$, ..., $u_{L-1}$ by analyzing the (arbitrarily chosen) leaf of the subtree rooted at $\overline{q}$ in every iteration of case 3. Moreover, we can retrieve:

- set $U_1$ of pairs $(i, \, u)$ such that $(u,u_2) \in E$ and $\alpha = \ell (u)[i..\Vert u \Vert ]$, by iterating over the leaves of $\overline{p}$;
- unique node $u_L$ such that $(u_{L-1},u_L) \in E$ and $\ell (u_L) = \beta$, if such $u_L$ exists; and
- set $E_L$ of triplets $(u,u',j)$ such that $(u_{L-1},u),(u,u') \in E$ and $\ell (u)\ell (u')[1..j] = \beta$.

Then $([1..y],(i, u_1 \cdot u_2 \cdots u_{L-1} \cdot u \cdot u_{L+1},j))$ is a MEM between *Q* and *G* for all $(i,u_1) \in U_1$ and $(u,u_{L+1},j) \in E_L$, and also $([1..y],(i, u_1 \cdot u_2 \cdots u_{L-1} \cdot u_L, \Vert u_L \Vert ))$ is a MEM for all $(i,u_1) \in U_1$, if $u_L$ exists: these MEMs satisfy conditions I, II, and III, and $U_1$, $u_2 \cdots u_{L-1}$, $u_L$, and $E_L$ form a compact representation of all MEMs spelling *Q*[1..*y*].

So far the procedure computes all MEMs spanning more than 3 nodes, satisfying $\textsf{LeftMax}$ and $\textsf{RightMax}$, and spelling maximal *Q*[1..*y*]. We can extend it to find all MEMs satisfying the first two constraints and spelling any substring *Q*[*x*..*y*], with *Q*[*x*..*y*] maximal. Let $\hat{x}$ be the index for which we have computed MEMs spelling $Q[\hat{x}..y]$ ($\hat{x} = 1$ in the first iteration). If cases 1 or 2 hold, we can start to search MEMs spelling $Q[\hat{x}+1..]$ in amortized linear time, since we follow the suffix link of $\overline{p}$. If case 3 holds, we can restart the algorithm looking for MEMs spelling $Q[\hat{x}'..y]$, where $\hat{x}' = \hat{x} + |\alpha \ell (u_2) \cdots \ell (u_{L-2}) |$. We are not missing any MEM satisfying conditions I, II, and III: due to the semi-repeat-free property, any MEM $([x..y],(i',P',j'))$ with $\hat{x}< x < \hat{x}'$ spanning more than 3 nodes shares substring $\ell (u_k)\ell (u_{k+1})$ with the previously computed MEM, for some $k \in [2..L-3]$, and is such that $\hat{x}' < y$ since we assume III to hold; the algorithm would have matched $Q[\hat{x}..y]$ with case 3 in the previous iteration, leading to a contradiction. The time globally spent reading *Q* is still $O(|Q |)$, because each character of *Q* is considered at most twice.

Finally, we are ready to describe how to compute all remaining MEMs ([*x*..*y*], (*i*, *P*, *j*)) between *Q* and indexable EFG *G* spanning at least 4 nodes, that is, MEMs such that condition I holds and at least one of II and III do not: it is easy to see that *Q*[*x*..*y*] must be contained in the MEMs that we have already computed; also, since they span at least 4 nodes their matches must involve some of nodes $u_2$, ..., $u_{L-1}$ of MEMs satisfying I, II, and III. Indeed, whenever case 3 holds and we decompose $Q[\hat{x}..y]$ as $\alpha \ell (u_2) \cdots \ell (u_{L-1}) \beta$, we can find set $U_{\text{RT}}$ of pairs (*v*, *j*), with $v \in V$ and $1 \le j \le \Vert v \Vert$, such that $(v,j) \in U_{\text{RT}}$ iff $(i, P = u \cdot u_2 \cdots u_{b-1} \cdot v, j)$ is a match of $Q[\hat{x}..y']$ in *G*, with $(i,u) \in U_1$, $|P |< L$, $y' < y$, and $Q[y'+1] \notin \mathop {\text{rext}}\limits (1,u,j)$—verifying $\textsf{RightMax}$ and describing a MEM where III fails—or $|\mathop {\text{rext}}\limits (1,u,j) |\ge 2$—verifying the non-singleton condition of Definition 1 and describing a MEM where II fails. We can gather all the elements of $U_{\text{RT}}$ during each descending walk in the suffix tree of $T_3'$, since they correspond to the leaves of subtrees of branching nodes in the tentative match of $Q[\hat{x}..y]$. Analogously, we can find set $U_{\text{LT}}$ of pairs (*i*, *v*), with $v \in V$ and $1 \le i \le \Vert v \Vert$, such that $(i,v) \in U_{\text{LT}}$ iff $(i,v) = (1,u_i)$ for $2 \le i \le L - 1$ and $\mathop {\text{lext}}\limits (u_i) \ge 2$, or $(i, P = v \cdot u_b \cdots u_{L-1}, \Vert u_{L-1} \Vert )$ is a match of $Q[x..y - |\beta |]$ in *G*, with $x > \hat{x}$ and $Q[x-1] \notin \mathop {\text{lext}}\limits (i,v,\Vert v \Vert )$. We can compute $U_{\text{LT}}$ by analyzing the leaves of subtrees of branching nodes in the walk of $T_{u_i}$ spelling $\overline{\ell (u_i)}$, with $2 \le i \le L-1$. Sets $U_1$, $u_2\cdots u_{L-1}$, $u_L$, $E_L$, $U_{\text{LT}}$ and $U_{\text{RT}}$ are a compact representation of all MEMs spanning at least 4 nodes and involving (any substring of) $Q[\hat{x}..y]$: a cross-product-like algorithm that matches elements of $U_1$ or *L* with elements of $u_L$, $E_L$, or $U_{\text{RT}}$, joined by the relevant part of $u_2 \cdots u_{L-1}$, can explicitly output the MEMs spanning more than 3 nodes in linear time with respect to the size of the output, by exploiting the fact that $U_{\text{LT}}$ and $U_{\text{RT}}$ are computed and ordered block by block.

### Theorem 1

Let alphabet $\Sigma$ be of constant size, and let $G=(V,E,\ell )$ be an indexable Elastic Founder Graph of height *H*, that is, the maximum number of nodes in a block of *G* is *H*. MEMs between query string $Q\in \Sigma ^m$ and *G* with arbitrary length threshold $\kappa$ can be reported in time $O(n H^2 + m +M_\kappa )$, where $n=\sum _{v\in V} \Vert v \Vert$ and $M_\kappa$ is the number of MEMs of interest.

### Proof

We can apply the algorithm of Lemma 3 to find $\kappa$-MEMs spanning *L* nodes, with $L = 1,2,3$, taking time $O(|Q |+ |T_1 |+ |T_2 |+ |T_3 |+ M_{\kappa ,1} + M_{\kappa ,2} + M_{\kappa ,3})$.

Let $M_{\kappa ,4+}$ be the number of MEMs satisfying threshold $\kappa$ and spanning at least 4 nodes in *G*. The suffix tree of $T_3'$ can be constructed in time $O(|T_3' |)$ and the described modification of a descending suffix walk on *Q* takes constant amortized time per step, assuming constant-size alphabet. The time spent gathering $U_1$, $u_2 \cdots u_{L-1}$, $E_L$, $U_\text{LT}$, and $U_{\text{RT}}$, forming an encoding of the MEMs involving $Q[\hat{x}..y]$, can be charged to $M_{\kappa ,4+}$ because each element of $U_1$, $E_L$, $U_\text{LT}$, and $U_{\text{RT}}$ corresponds to one or more MEMs, that could be retrieved in an explicit form with a cross-product-like procedure. Indeed: for $U_1$ we can retrieve all leaves of the subtree rooted at $\overline{p}$ of the suffix tree of $T_3'$; for $E_L$ and $U_{\text{RT}}$, we can do the same for node $\overline{r}$ reached by the last tentative match of $Q[y+1..]$, and for branching nodes reached during every tentative match; for $U_\text{LT}$, using a compact trie and *blind search* [41] in the representation of each $T_u$ allows to compare only the branching symbols. Finally, it is easy to see that in case 14, after we decomposed $|Q[\hat{x}..y] |$ as $\alpha \ell (u_2) \cdots \ell (u_{L-1}) \beta$ as in Observation 1, we know the length of strings $\alpha$, $\ell (u_2)$, ..., $\ell (u_{L-1})$, and $\beta$, so we can postpone the computation of sets $U_1$, $E_L$, $U_\text{LT}$, and $U_\text{RT}$ and avoid computing MEMs of length smaller than $\kappa$. Thus, finding an encoding of all MEMs between *Q* and *G* with threshold $\kappa$ and spanning more than 3 nodes takes $O(|Q |+ |T_3' |+ M_{\kappa ,4+})$ time.

The stated time complexity is reached due to the fact that $|T_3 |$ dominates $|T_3' |$, $|T_2 |$, and $|T_1 |$, and for indexable EFGs $|T_3 |\in O(n H^2)$, since every character of every node label $\ell (u)$ gets repeated at most $H^2$ times, which is an upper bound on the number of paths of length 3 containing *u*.

### Corollary 1

(batch queries) The results of Lemmas 2 and 3 and Theorem 1 hold when query *Q*[1..*m*] is replaced by a set of queries of total length *m*. The respective algorithms can be modified to report MEMs between the graph and each query separately.

### Proof

Consider a concatenation $Q=Q^1\$ Q^2\$ \cdots Q^d$ of *d* query sequences, where ${\$}$ is a unique symbol not occurring in the queries nor in the graph. No MEM can span over the unique separators and hence the MEMs between graph *G* and concatenation *Q* are the same as those between *G* and each $Q^i$. It is thus sufficient to feed concatenation *Q* as input to the algorithms and project each resulting MEM to the corresponding query sequence.

### Corollary 2

(filtering) The algorithms of Lemmas 2 and 3, Theorem 1, and Corollary 1 can be modified to report only MEMs that occur in text *T* formed by concatenating the rows (ignoring gaps and adding separator symbols) of the input MSA of the indexable EFG. This can be done in additional $O(|T|+r\log r)$ time and $O(r\log n)$ bits of space and with multiplicative factor $O(\log \log n)$ added to the running times of the respective algorithms, where *r* is the number of equal-letter runs in the BWT of *T*.

### Proof

Lemma 2 does not need any modification as the node labels are automatically substrings of *T*. The same applies to the edge labels, but for longer paths, we need to make sure we do not create combinations not supported by *T*. This can be accomplished with the help of the *r*-index: with the claimed time and space one can build the run-length encoded BWT of *T* [42] and the associated data structures to form the counting version of the *r*-index that supports backward step in $O(\log \log n)$ time [43]. As we concatenate paths consisting of *L* nodes for MEM finding in Lemma 3, we can first search them using the *r*-index and only include them if they occur in *T*. MEMs spanning more than 3 nodes in Theorem 1 and Corollary 1 can be searched afterward with the *r*-index to filter out those MEMs not occurring in *T*; these MEMs cannot mutually overlap each other in *Q* by more than one full node label, so the running time of the verification can be charged on the size of the Elastic Founder Graph.

### Corollary 3

(indexing) Running times in Lemmas 2 and 3 can be separated to an *O*(|*T*|)-time indexing phase and $O(|Q|+N_{\texttt{rmax}}+\texttt{occ})$-time query phase, assuming constant alphabet. Here $N_{\texttt{rmax}}\le \min (|Q |^2, |Q |+ |T |)$ is the number of recursion tree nodes visited and $\texttt{occ}$ is the number of $\kappa$-MEMs reported by the respective algorithm.

### Proof

Indexing *T* and *Q* was shown to take linear time. Exploring the MEM candidates takes $O(N_{\texttt{rmax}})$ time. The cross-product routine is output-sensitive on constant alphabet $\Sigma$. It was shown that $N_{\texttt{rmax}}\le |Q|+|T|$. Since each MEM candidate $Q'$ needs to be a substring of *Q* and the algorithm makes at most $|\Sigma |$ left-extensions from $Q'$ that leads to $aQ'$ that does not occur in *Q*, it follows that $N_{\texttt{rmax}}\le |\Sigma ||Q|^2$.

## Experiments

### Comparison to r-index

The benefit of Corollary 2 over the mere use of *r*-index for MEM finding [44] is that a MEM can occur many times in a repetitive collection while the occurrences starting at the same column of an MSA of the collection can be represented by a small number of paths in the indexable Elastic Founder Graph.

To test this hypothesis, in the conference version of this paper [26] we implemented the MEM finding algorithm using the bidirectional r-index [25]. That implementation covers the algorithms described earlier up to paths of length 3 nodes. Although we were able to demonstrate that the number of MEMs on the graph is significantly smaller than on the concatenation of sequences, the scalability of the approach was not satisfactory: while node and edge concatenations yield competitive space and time, paths of length 3 are a bottleneck [26].

**Fig. 3:**
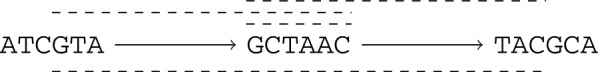
A $\kappa$-MEM (dashed line on the bottom) split to full node MEM, edge suffix MEM, and edge prefix MEM (dashed lines on the top)

To make the approach scalable, we engineered the approach further so that paths of length 3 are no longer required. Namely, we observed that it suffices to consider *full node MEMs* and *edge prefix and suffix MEMs*: Full node MEMs are such that $Q[i..j]=\ell (v)$ for some node *v*. Edge prefix MEMs are edge MEMs s.t. $Q[i..j]=\ell (u)\ell (v)[1..j']$, where (*u*, *v*) is an edge in the graph and $j'\ge 1$. Edge suffix MEMs are edge MEMs s.t. $Q[i..j]=\ell (u)[i'..|\ell (u)|]\ell (v)$, where (*u*, *v*) is and edge in the graph and $1\le i'\le \ell (u)$. While we consider minimum length $\kappa$ in node and edge MEMs, we do not restrict the length of full node, edge prefix, and edge suffix MEMs. This has the negative consequence that we lose the theoretical guarantee on the number of MEMs reported. However, we do not lose the guarantee on the accuracy of the approach: A MEM of length at least $\kappa$ that does not occur inside a node or inside an edge can be split into a (possibly empty) suffix edge MEM, one or more full node MEMs, and a (possibly empty) prefix edge MEM (see Fig. 3). That is, the set of $\kappa$-MEMs is fully represented by node $\kappa$-MEMs, edge $\kappa$-MEMs, full node MEMs, and edge prefix and suffix MEMs. For the computation of the latter, it is sufficient to use the same index as for edge $\kappa$-MEMs. In fact, the algorithm to compute these is simpler: For full node MEMs, one can just backward search $\ell (v)$ for all nodes *v* in the BWT index of the query *Q*. For reporting edge suffix MEMs for edge (*u*, *v*) one can proceed similarly backward searching $\ell (u)\ell (v)$ using the BWT index of *Q*. After having read $c\ell (v)$, where $c=\ell (u)[|\ell (u)|]$, we check if the range shrinks with the next symbol, $d=\ell (u)[|\ell (u)|-1]$, and report the occurrences $Q[i..j]=c\ell (v)$ such that $Q[i-1]\ne d$ or $i=1$. This reporting can continue until $\ell (u)\ell (v)$ is read or the search range becomes empty. The reporting of edge prefix MEMs is symmetric. Furthermore, if we start reporting MEMs just after reaching the node boundary, the latter algorithms also report full node MEMs, so we do not need to report them separately.

In the following, we repeat the same experiment as in the conference version but we replace path MEMs with full node MEMs and edge suffix and prefix MEMs.

We performed experiments with the same multiple sequence alignment (MSA) of SARS-CoV-2 strains as in [45]. We first filtered out strains whose alignments had a run of gaps of length of more than 100 bases.^2^ Then we extracted a sub-MSA of 100 random strains from the remaining and extracted MSAs of the first 20, 40, 60, and 80 strains from this MSA of 100 strains. For each such dataset, we built the bidirectional r-index of the sequences (without gaps) and the indexable EFG of the MSA. The latter was constructed using the tool https://github.com/algbio/founderblockgraphs with parameters—elastic—gfa. We post-processed the resulting GFA file by merging unary paths, as this merging does not break the semi-repeat property. In this merging, we left the first and the last nodes of a unary path unaltered to minimize the growth of edge concatenations.

We used $\kappa =12$ in all experiments: this parameter was chosen in order to be backward-compatible with the experiments on the previous version of the tool [26]. For the queries, we extracted 1000 substrings of length 100 from the first 20 strains. For each query, we selected two random positions and mutated them with equal probability for A, C, G, or T. The queries were then concatenated into a long sequence and the bidirectional r-index was built on it as described by Corollary 1. The MEMs were computed between the queries and the respective text/graph index.

**Fig. 4:**
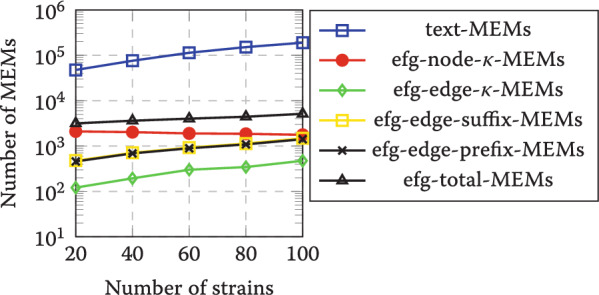
Number of MEMs with different indexes and varying number of SARS-CoV-2 strains. Here, text-MEMs refers to bidirectional r-index. For indexable EFG the results are shown for node $\kappa$-MEMs, edge $\kappa$-MEMs, edge suffix MEMs, and edge prefix MEMs. Line efg-total-MEMs is the total number of EFG MEMs. Note the logarithmic scale on the *y*-axis

**Fig. 5:**
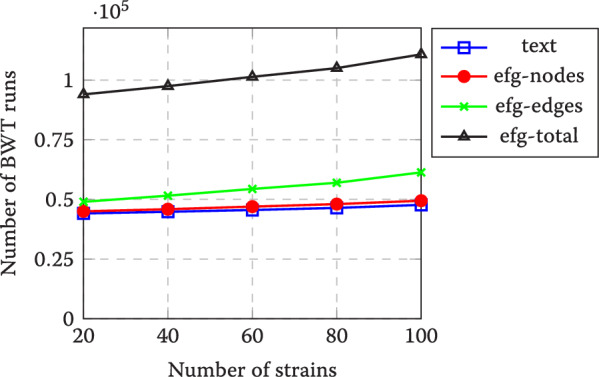
Number of BWT runs with different indexes and varying number of SARS-CoV-2 strains. Here text refers to the bidirectional r-index, while labels efg-nodes and efg-edges refer to EFG node concatenations and edge concatenations, respectively. Label efg-total is the sum of the previous two numbers of runs

The number of MEMs for each index is reported in Fig. 4 and the number of runs in the two Burrows-Wheeler transforms of each index is reported in Fig. 5. As can be seen from the results, the number of MEMs is greatly reduced when indexing the graph compared to indexing the collection of strains. Moreover, long MEMs may be reported in smaller pieces, so the actual number of $\kappa$-MEMs can be much smaller than what is reported.

The number of runs (a major factor affecting the space used by the indexes) for the bidirectional r-index of the collection and that of the concatenation of node labels are comparable. For edges the number of runs is slightly higher. Fortunately, the growth of these metrics when more strains are added is limited. This is not surprising, as the strains are highly similar and thus the added information content is limited and known to be correlated with the number of BWT runs [46].

Table 1 gives resource usage statistics for the largest collection of strains. The running times and space usages were measured on a server with Intel Xeon 2.9 Ghz processor and include index construction. Resident Set Size (RSS) was used as the measurement tool. To speed up MEM finding on indexable EFGs, we also tested switching the underlying bidirectional r-index to a wavelet tree implementation of the bidirectional BWT (https://www.github.com/jnalanko/BD_BWT_index). As can be seen, this gives a 9-fold speed up over the use of bidirectional r-index with EFG. The space bottleneck on the bidirectional r-index is the indexing part, while with both EFG implementations it is the MEM finding part. The former used 7.7 MB during MEM finding, which is less than the EFG implementations did.

**Table 1:** Input data and EFG properties (top)

Text	Length (bases)	Number of runs in forward BWT	Number of runs in reverse BWT
100 strains of SARS-Cov-2	2,978,342	23,856	23,848
EFG node concatenation	42,379	24,869	24,890
EFG edge concatenation	85,909	30,653	30,655

Index	Time (seconds)	Space (MB)	
bidirectional r-index	21	52.5	
EFG using bidirectional r-index	226	14.7	
EFG using bidirectional BWT index	25	11.6	

Time and space usage of MEM finding on different indexes (bottom)

### Indexing properties

**Fig. 6:**
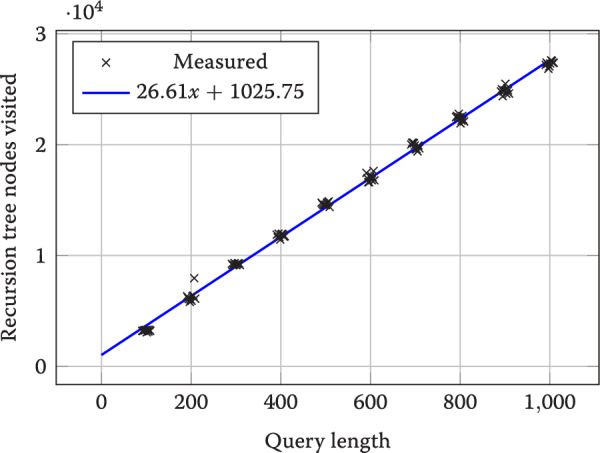
The number of recursion tree nodes for efg-node-$\kappa$-MEMs grows linearly by the query length (data shown against the best fit linear function). Ten different queries per length were measured. We added a small offset in the x-axis to show that there are indeed many measurements, but the values are very close

While our MEM finding approach has been designed for batch processing, we wanted to check if it can be used as an index. As considered earlier, the worst-case query time is quadratic in the query length, but it is not clear if this worst case is achieved in practice. To test this, we created a multiple alignment of *E. coli* reference genome and 9 mutated variants of it. Each of these mutated variants was created by choosing uniformly at random 1% of the bases and mutating these bases with 1/3 probability to one of the other bases. Then we constructed the indexable EFG of this MSA and created queries of length $100, 200, \ldots , 1000$ by sampling from the reference as in the earlier experiment and mutating 2% of bases. Figure 6 plots the number of recursion tree nodes visited when exploring $\kappa$-MEMs on node concatenations with 10 queries over each different length. There appears to be a linear dependency on the query length and only a small deviation within each query length, so the average running time may be better than the worst case.

## Conclusions

Motivated by co-linear chaining of matches between a sequence and a labeled graph, we studied the problem of finding MEMs of at least length $\kappa$ between a sequence and a graph, we proposed an efficient solution to compute $\kappa$-MEMs spanning 1 node, and we generalized this computation to MEMs spanning *L* nodes and to indexable Elastic Founder Graphs. As explained in the introduction, $\kappa$-MEM finding on graphs admits a quadratic-time conditional lower bound, so this naturally poses the question of whether a quadratic-time solution exists. We believe that a quadratic-time solution to $\kappa$-MEM finding on graphs can be achieved with an application of the *labeled direct product* [22]—the graph based on the Cartesian product that finds all subpaths of *G* and substrings of *Q* spelling the same string—and we reserve the study of this problem as future work.

An alternative strategy to achieve the same goal as in our experiments is to encode the graph as an aggregate over the collection, apply MEM finding on the r-index, and report the distinct aggregate values on lexicographic MEM ranges to identify MEM locations in the graph [47]. This approach is not comparable to ours directly, as the compressibility of the aggregates depends on the graph properties, and the indexable Elastic Founder Graph’s size has not been analyzed with respect to *r*. Also, the two approaches use different MEM definitions. Our Definition 1 is symmetric and local, while the version used in earlier work with the *r*-index [44, 47] is asymmetric and semi-global: they define a MEM as a substring of a query that occurs in the text, but its query extensions do not appear in the text. For the purpose of chaining, only the symmetric definition yields connections to the Longest Common Subsequence problem [38]. For completeness, our implementation also supports this asymmetric MEM definition; our algorithms can be simplified for this case.

We did not implement the general suffix tree-based approach to handle arbitrary long MEMs, but instead a relaxed variant that does work correctly, while unfortunately sacrificing output-sensitivity. In our recent work [39], we have solved pattern search in indexable EFGs using only edges, and our aim is to extend that approach to work with MEMs, so that the whole mechanism could work on top of a plain bidirectional r-index, without resorting to relaxation. Also, our implementation has not been optimized for space usage, and therefore we studied an implementation-independent indicator, the number of runs in the BWT, in more detail. This is to illustrate the potential of the approach. Currently, the implementation uses linear space with respect to the length of the edge concatenations rather than to the number of runs in its BWT. It is easy to modify the implementation to be linear in the number of BWT runs, trading the space of suffix array and range minimum queries on the *D*-array to reporting duplicate MEMs. We leave it for future work to experimentally evaluate this option.

Finally, all of our theoretical results assume a constant-size alphabet. This assumption can be relaxed with additional data structures. We are working on a more careful amortized analysis to relax such assumption.

## Data Availability

The code and data to reproduce the experiments are available in https://github.com/algbio/efg-mems.

## Footnotes

1 Note that Equi et al. assume *G* to be a node-labeled graph with labels of length 1, i.e. $\ell : V \rightarrow \Sigma$, whereas in this paper we assume the labels to be non-empty strings over alphabet $\Sigma$, i.e. $\ell : V \rightarrow \Sigma ^+$. The latter class of graphs includes the former, so the quadratic-time lower bound still holds.

2 We used this filter to address a current limitation of the MSA segmentation algorithms for indexable EFG, where a long gap at the beginning or end of an MSA row hurts the effectiveness of the method.
